# Monitoring the Brain’s Response to Alcohol With Positron Emission Tomography

**Published:** 1995

**Authors:** Nora Volkow, Gene-Jack Wang, John J. Doria

**Affiliations:** Nora Volkow, M.D., is a scientist at Brook-haven National Laboratory and associate professor at the Department of Psychiatry, State University of New York, Stony Brook. Gene-Jack Wang, M.D., is a scientist at Brookhaven National Laboratory, Upton, New York. John J. Doria, M.S., is a science editor of Alcohol Health & Research World

**Keywords:** brain, AODE (alcohol and other drug effects), positron emission tomography, blood circulation, metabolism, radionuclide imaging, AOD abstinence, AOD impairment, neurotransmission, hereditary factors

## Abstract

Researchers have used positron emission tomography (PET) to study the interrelationships of structural, metabolic, and functional brain changes following alcohol consumption as well as during withdrawal and abstinence. This technique is based on the fact that blood flow and energy metabolism tend to increase in parts of the brain undergoing increased activity and to decrease in brain tissue that is diseased or damaged. PET detects such changes by tracking the distribution within the brain of radioactive chemicals that have been injected into the blood. Because of its sensitivity, PET can detect early functional deficits in the brain before structural changes are apparent. PET has documented gradual recovery of cognitive functions with continued abstinence. In addition, PET data has been used to investigate possible mechanisms for some of alcohol 3s effects and to provide additional evidence for the heritability of alcoholism.

Alcoholism^1^ is associated with a spectrum of cognitive dysfunctions ranging from severe dementing syndromes to more common, subtle disturbances of information processing, problem-solving, and memory (Porjesz and Begleiter 1983; Malloy 1992; Charness 1993). Measurements of blood flow and energy metabolism in the brain are sensitive indicators of such brain function (Volkow and Tancredi 1991). These measurements have been used to identify specific brain regions affected by alcohol and to investigate mechanisms of alcohol-induced cognitive and behavioral impairment.

Researchers have obtained these measurements using various imaging methods, including magnetic resonance imaging (MRI) techniques and single-photon emission computed tomography (SPECT). These methods are described in Mazziotta (1994) and Pfefferbaum and Rosenbloom (1993) and in the article by Doria, pp. 261–265. This article discusses the use of positron emission tomography (PET) to confirm and extend the results of other imaging studies on the interrelationships of structural, metabolic, and functional brain changes following both brief (i.e., acute) and long-term (i.e., chronic) alcohol consumption as well as during withdrawal and abstinence. In addition, the article uses PET data to explore a possible mechanism for some of alcohol’s effects to support the heritability of alcoholism.

## Monitoring Brain Function With PET

Blood flow and energy metabolism tend to increase in parts of the brain undergoing increased activity and to decrease in brain tissue that is diseased or damaged. PET detects these changes by tracking the distribution within the brain of chemicals known as tracers, which are injected into the blood. These tracers are rendered radioactive (i.e., radiolabeled) by being chemically combined with radioactive variants (i.e., radioisotopes) of elements found naturally in the human body. Special cameras surrounding the subject’s head track the radioactive tracer as it moves through the brain’s bloodstream. A computer translates the resulting data into color-coded images.

PET has had two major applications to date. First, PET can measure blood flow for the entire brain (i.e., cerebral blood flow [CBF]) or for specific regions within the brain (regional CBF [RCBF]). Second, PET can measure brain energy metabolism by tracking the distribution of the radiolabeled tracer fluorodeoxyglucose (FDG), a variation of glucose. Because glucose is normally the brain’s sole energy source, the distribution of FDG in the brain reflects relative energy metabolism in different brain regions.

## Blood Flow and Brain Function

Acute exposure to alcohol (e.g., a single drinking session) may produce brain impairments ranging from visible signs of intoxication (e.g., staggering gait) to decreased performance in specialized tests of cognitive function. Many tracer studies using techniques other than PET have reported decreased CBF in alcoholics (for example, Melgaard et al. 1990; Rogers et al. 1983). Using PET, Volkow and colleagues (1988) extended these studies to relate alcohol’s CBF effects to muscular incoordination and self-ratings of intoxication in nonalcoholic men. Low doses were adjusted to 0.5 grams of alcohol per kilogram of body weight (g/kg) (i.e., approximately three drinks), and moderate doses were adjusted to 1.0 g/kg (approximately seven drinks). Blood flow was measured before alcohol administration and at both 40 and 60 minutes after administration. The most significant effect was a reduction of blood flow to the cerebellum under the higher dose.

The cerebellum, located at the base of the brain, is involved primarily in postural stability and motor coordination. Therefore, decreased cerebellar blood flow is consistent with the disruption of muscular coordination observed in intoxicated people. The prominence of this effect suggests that the cerebellum may be one of the brain areas most sensitive to alcohol.

In the bulk of the brain (i.e., the cerebrum), the higher of the two alcohol doses increased the RCBF in both the prefrontal cortex and the right temporal cortex (Volkow et al. 1988). The increased RCBF in these regions was most pronounced after 60 minutes, when the blood alcohol concentration peaked. The increased CBF in these cortical areas may be related to the general mood activation of the subjects, who became euphoric and more talkative after consuming the alcohol; Tiihonen and colleagues (1994) subsequently reached a similar conclusion using SPECT.

Alcohol’s tendency to increase blood flow in the right, rather than the left, temporal cortex is consistent with results of neuropsychological and electroencephalographic (i.e., “brain wave”) tests in which alcohol predominantly affected the functions of the right half of the cerebrum (Porjesz and Begleiter 1975).

## Brain Energy Metabolism

The constriction and dilation of blood vessels caused by alcohol make it difficult to assess the relation between CBF data and brain function (Newlin 1982; Altura and Altura 1987). Conversely, metabolic measures of brain function are easier to interpret because they reflect brain cell activity directly, independently of blood vessel responses.

**Figure 1:**
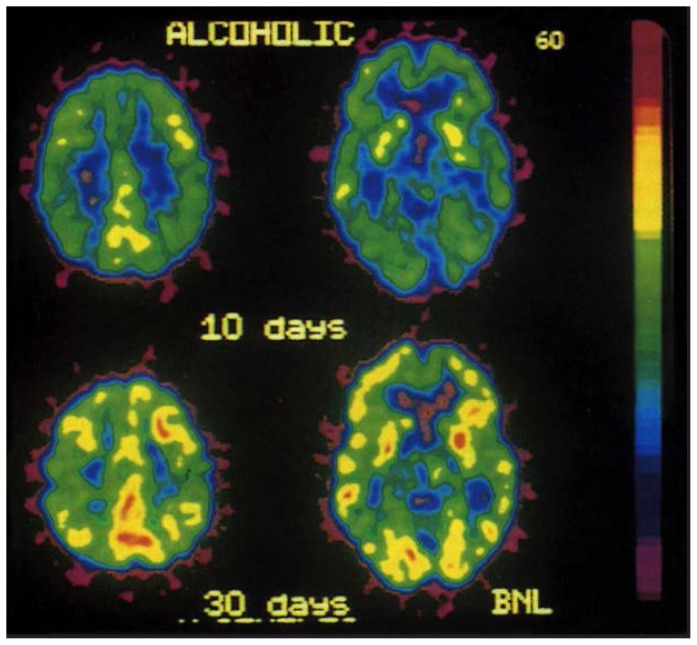
Positron emission tomography brain images in an alcoholic subject showing increases in brain metabolism between 10 and 30 days after cessation of alcohol consumption. SOURCE: Volkow et al. 1994.

Volkow and colleagues (1990) tested six nonalcoholic and six alcoholic subjects using PET and FDG before and 24 hours after subjects consumed 1 g/kg of alcohol. Alcohol decreased energy metabolism in the cerebral cortex and the cerebellum in both the alcoholics and the nonalcoholics (figure 1), but more so in the alcoholics. In addition, the researchers confirmed that the decreased brain energy metabolism did not result from alcohol’s effect on blood vessels, because acute alcohol intoxication *increased* CBF in the frontal cortex as it decreased frontal metabolism (see also de Wit et al.1990).

## Brain Metabolism, Structure, and Function

Structural changes observed in the brains of alcoholics include a decrease in brain size as indicated by cortical atrophy (i.e., shrinkage) and enlargement of the ventricles (i.e., fluid-filled cavities within the brain) (Zakhari and Witt 1992). The significance of these structural changes to brain function is poorly understood. To assess the relationship among brain structural changes, metabolism, and function, Wang and colleagues (1993) compared 10 alcoholic with 10 nonalcoholic subjects, using PET to measure regional brain energy metabolism. The researchers used MRI to measure both cortical atrophy and ventricular size between 6 and 32 days after the alcoholic subjects last consumed alcohol. The alcoholics were found to have decreased brain energy metabolism and more cortical atrophy compared with the nonalcoholics. No difference was found in ventricular size between the two groups. The degree of ventricular enlargement and cortical atrophy was associated with decreased metabolism, particularly in the frontal region of both the alcoholics and the nonalcoholics.^2^ Brain structural changes were unrelated to brain functions assessed by performance on neuropsychological tests. However, frontal lobe metabolism was correlated with performance on specific subtests relating to planning and short-term memory. The researchers concluded that PET with FDG is a sensitive technique for detecting early functional brain deficits before structural changes become apparent using MRI.

## Withdrawal and Recovery

The alcoholic subjects in the studies cited above were not actively drinking. This raises the question of brain structure and function during the phases of withdrawal and recovery from alcoholic drinking episodes. Many researchers have used PET to investigate withdrawal and recovery in alcoholics, but studies have yielded conflicting results. Two PET studies reported decreased brain energy metabolism (Sachs et al. 1987; Wik et al. 1988), whereas another PET study reported no change (Samson et al. 1986). One factor that might account for these discrepancies is the time during withdrawal at which the studies were performed.

Volkow and colleagues (1994) evaluated metabolic recovery using PET and FDG in 10 male alcoholics who were tested three times within a period of 8 to 60 days after last using alcohol. During the initial evaluation, the alcoholics manifested significantly lower regional metabolism compared with nonalcoholic control subjects, predominantly in the frontal and parietal cortices. Brain metabolism increased significantly within 16 to 30 days, especially in the frontal regions. Older alcoholic subjects and those with longer histories of alcohol use exhibited lower brain metabolic rates compared with the control subjects.

By 60 days after last consuming alcohol, the alcoholics’ brain metabolic rates were comparable to those of the control subjects except for persistently low metabolic rates in the alcoholics’ basal ganglia. Significantly, no patients in the study exhibited abnormal movements (e.g., tremors) typical of basal ganglia disorders, although such movements may occur transiently during alcohol withdrawal (Neimann et al. 1990).

Additional experiments (Volkow et al. 1992) have determined that the metabolic changes documented in withdrawing alcoholics occur in the absence of signs or symptoms of withdrawal^3^ and without evidence of major cognitive impairment based on neuropsychiatric tests.

## Alcohol Affects Nerve Cell Communication

All brain functions involve communication among nerve cells, which in turn requires the expenditure of energy. Thus, the energy metabolism measured by PET in a given region reflects to some extent the level of communication among local nerve cells as they perform their specific functions. Communication between adjacent nerve cells generally involves the release of chemical messengers called neurotransmitters. A message is “received” when a neurotransmitter temporarily attaches, or binds, to its receptor protein in the surface of a nerve cell.

The neurotransmitter gamma-aminobutyric acid (GABA) regulates brain function by inhibiting nerve cell communication. Research suggests that GABA may contribute to the intoxicating effects of alcohol and the development of alcoholism (Ticku and Burch 1980; Ticku et al. 1983; Littleton 1989; Grant 1994). Therefore, Volkow and colleagues (1993) used PET and FDG to evaluate whether the function of the GABA system differs between alcoholics and nonalcoholics (Volkow et al. 1993). The researchers administered a dose of the sedative lorazepam (Ativan^®^), known to increase the effects of GABA, to 12 nonalcoholic and 10 recently detoxified alcoholic subjects. Lorazepam decreased whole brain energy metabolism in both groups.

The nonalcoholic and alcoholic subjects showed similar responses to lorazepam in occipital and cerebellar metabolism, whereas the alcoholic subjects exhibited significantly less of a response (i.e., a blunted response) to lorazepam in the thalamus, basal ganglia, and orbitofrontal cortex. The blunted metabolic response to lorazepam in alcoholics is consistent with decreased GABA receptor function. Analysis of the data suggests that blunting of the effects of lorazepam in these specific brain regions in alcoholics may be a consequence of alcohol-related changes in cerebellar metabolism (Volkow et al. 1993). Such changes might interrupt the inhibitory signals that normally flow from cerebellar nerve cells to those structures (Linas and Watton 1990; Brodal 1981; Harper and Heath 1973). Significantly, these structures form part of a communication circuit regulating the initiation and termination of behaviors. Additional research might determine if any relation exists between decreased inhibitory control of these regions and the lack of control over drinking that characterizes alcoholism (Modell et al. 1989).

## Genetics: Subjects at Risk

The blunted response of brain metabolism to lorazepam in alcoholics may result from chronic alcohol use. Alternatively, the blunted response may represent a genetic trait associated with preexisting vulnerability to alcoholism. For example, children of alcoholics (i.e., family history-positive subjects [FHP’s]) show a blunted response to various signs and symptoms of alcohol administration compared with subjects with no family history of alcoholism (i.e., family history-negative subjects [FHN’s]) (Begleiter et al. 1984; Schuckit et al. 1983, 1991; Porjesz and Begleiter 1983). The GABA receptor may play a part in this differential sensitivity to alcohol’s effects (Allan and Harris 1991).

A study by Cowley and colleagues (1994) supports this hypothesis. The FHP’s and FHN’s were compared in terms of their response to diazepam (Valium^®^), a sedative belonging to the same class as lorazepam (e.g., a benzodiazepine). Both groups of subjects received increasing doses of diazepam (25 to 100 micrograms per kilogram [μg/kg]) or a placebo on 2 days at least 1 week apart. The researchers assessed the effects of diazepam using two eye-movement tasks that objectively measured benzodiazepine effects. The FHP’s displayed significantly less diazepam effects on eye movements than the FHN’s. Effects increased as the dose increased.

Volkow and colleagues (1995) measured the effects of 30 μg/kg of lorazepam on regional brain energy metabolism using PET and FDG in both FHP’s and FHN’s. At baseline (i.e., before drug administration), the FHP’s showed lower cerebellar metabolism than the FHN’s. Although lorazepam decreased whole-brain energy metabolism in both the FHP’s and FHN’s, this response was blunted in the cerebellum of the FHP’s. The decreased cerebellar metabolism at baseline in the FHP’s, together with the blunted cerebellar response to lorazepam administration in these same subjects, may reflect dysfunction of GABA receptors in the cerebellum. These changes could account for the decreased sensitivity to the effects of alcohol and benzodiazepines on motor function in the FHP’s.

## Summary

Researchers have used PET measurements of blood flow and energy metabolism to identify brain regions affected by alcohol and to investigate mechanisms of alcohol-induced cognitive and behavioral impairment. Because of its sensitivity, PET can detect early functional deficits in the brain before structural changes can be detected. Thus, PET studies have shown that alcohol withdrawal changes regional brain metabolism in alcoholics in the absence of neurological or psychological impairment. PET has also documented gradual recovery of cognitive functions with continued abstinence.

The cerebellum appears to be one of the areas of the brain most sensitive to alcohol. Alcohol-related changes in cerebellar metabolism might help explain the lack of control over drinking associated with alcoholism. Cerebellar metabolism and drug response, as revealed by PET, suggest that abnormal GABA receptor function may help characterize subjects with family histories of alcoholism.

## Footnotes

1 Because an overlap exists in the use of the term “alcoholism” in the literature, the term is used in each case based on its use in the reference cited.

2 Some degree of brain shrinkage is expected among nonalcoholic populations, especially as a result of aging.

3 Withdrawal symptoms may include sweating, nausea, vomiting, elevated blood pressure, and convulsions.
